# Prediction of Cyclin-Dependent Kinase Phosphorylation Substrates

**DOI:** 10.1371/journal.pone.0000656

**Published:** 2007-08-01

**Authors:** Emmanuel J. Chang, Rashida Begum, Brian T. Chait, Terry Gaasterland

**Affiliations:** 1 Department of Chemistry, York College of the City University of New York, Jamaica, New York, United States of America; 2 Laboratory of Mass Spectrometry and Gaseous Ion Chemistry, The Rockefeller University, New York, New York, United States of America; 3 Scripps Institute of Oceanography, University of California at San Diego, La Jolla, California, United States of America; University of Glasgow, United Kingdom

## Abstract

Protein phosphorylation, mediated by a family of enzymes called cyclin-dependent kinases (Cdks), plays a central role in the cell-division cycle of eukaryotes. Phosphorylation by Cdks directs the cell cycle by modifying the function of regulators of key processes such as DNA replication and mitotic progression. Here, we present a novel computational procedure to predict substrates of the cyclin-dependent kinase Cdc28 (Cdk1) in the *Saccharomyces cerevisiae*. Currently, most computational phosphorylation site prediction procedures focus solely on local sequence characteristics. In the present procedure, we model Cdk substrates based on both local and global characteristics of the substrates. Thus, we define the local sequence motifs that represent the Cdc28 phosphorylation sites and subsequently model clustering of these motifs within the protein sequences. This restraint reflects the observation that many known Cdk substrates contain multiple clustered phosphorylation sites. The present strategy defines a subset of the proteome that is highly enriched for Cdk substrates, as validated by comparing it to a set of *bona fide*, published, experimentally characterized Cdk substrates which was to our knowledge, comprehensive at the time of writing. To corroborate our model, we compared its predictions with three experimentally independent Cdk proteomic datasets and found significant overlap. Finally, we directly detected *in vivo* phosphorylation at Cdk motifs for selected putative substrates using mass spectrometry.

## Introduction

The reversible modification of proteins by covalent addition and removal of phosphate is a major means by which cellular function is regulated [1], [2]. The addition of phosphate, which is a sterically bulky and negatively charged moiety, can alter a protein's biochemical properties and affect its structure and activity. For example, phosphorylation can create docking sites to mediate protein interactions [2], modify signal sequences on proteins to regulate their subcellular localization [3], or activate enzymes by bringing their active sites into proper alignment [4]. Networks of phosphorylation-induced signaling can result in complex effects such as signal amplification, feedback inhibition or induction of cyclical oscillation between different cellular states [5]–[8]. Therefore, a computational tool that accurately predicts phosphorylation events could contribute to a more complete understanding of cell function [9].

Phosphorylation prediction algorithms must select, from all amino acid sequence space, a subset of amino acid sequences that are able to interact with one or more kinases as phosphate acceptors. The somewhat limited success of current phosphorylation prediction algorithms likely arises from the very large number and variety of both kinases and potential phosphate acceptors (Ser, Thr and Tyr residues) [2], [10]. A major difficulty in protein phosphorylation prediction stems from the fact that each kinase has its own particular specificity determinants [11], [12]. In some cases a particular kinase may require its substrate to have a highly stringent recognition site, whereas other kinases may be relatively promiscuous. Other kinases require restraints that may be distal to the recognition site, or consensus motif. Furthermore, it is possible that in certain cases, different kinases may have partially overlapping specificity, so that a single acceptor residue can be phosphorylated by more than one kinase. The challenge in developing a phosphorylation prediction tool is to effectively model molecular recognition mechanisms between individual kinases and their substrates, where the mechanisms can vary broadly for different kinases, and for which little experimental data may be available.

Most current strategies for the prediction of phosphorylation sites model the amino acid sequence (or a so-called consensus motif), which represents a kinase-specific phosphorylation site. Proteins that contain an instance of a given kinase's consensus motif are predicted to be substrates of that kinase. The simplest example of this type of strategy is linear motif searching, using computational tools such as PROSITE [13] and ELM [14]. This type of strategy searches for instances of phosphorylation consensus motifs represented by regular expressions. Other algorithms, such as ScanSite and PHOSITE utilize position-specific profile searches, which allow for more flexible definitions of consensus motifs [15]–[17]. Machine learning approaches, (*e.g.* hidden Markov models [18]–[20] and artificial neural networks [9], [21], [22]) have been used to model interdependencies between amino acids within a given consensus motif. NetPhos and NetPhosK are leading methods for phosphorylation site prediction that utilize artificial neural networks. Certain other procedures such as PREDIKIN utilize three-dimensional structural modeling to try to predict kinase-specific phosphorylation [23].

Attempts to evaluate the effectiveness of any phosphorylation prediction method face a two-fold difficulty. First, since the complete set of phosphorylation sites on all proteins is not known, it is not possible to assess the comprehensiveness of phosphorylation prediction. Therefore, both “false” positive and “true” negative designations may actually be assigned incorrectly to *true* phosphorylation sites that have yet to be discovered. Second, since only a limited number of sites are known for many kinases, it is likely that the known set of phosphorylation sites for a particular kinase is systematically biased and that any given algorithm may be unwittingly designed or trained to miss true positives. The most reliable measure of confirmation of phosphorylation-site prediction is the identification of such sites as *bona fide in vivo* phosphorylation sites through experiment. Because this is often laborious and not straightforward, few broad-based computational phosphorylation prediction procedures have had their results substantially confirmed through experimental verification.

### Strategy

One hallmark of nearly all published phosphorylation prediction procedures is that they employ a strategy to model the substrate specificity for as many kinases as possible. Such tools, which utilize the same strategy for different kinases, may inadvertently miss elements of substrate recognition that are in some way unique to a particular kinase system. Here, rather than developing a general model to predict substrates for many kinases, we instead propose a targeted procedure that models the substrate specificity in a more detailed way for a single well-studied family of kinases, *i.e.*, the cyclin-dependent kinases (Cdks) [5], [24], [25]. By targeting a single family of kinases, we should be better positioned to consider discriminating factors specific to this family. Thus, in the present procedure we are able to incorporate additional global characteristics that occur specifically between Cdks and their substrates, introducing a second factor that are not considered when modeling only local sequence motifs.

Cdks are the master regulators of eukaryotic cell cycle progression, coordinating events such as DNA synthesis and mitosis that are necessary for proper cell division and driving the cell-division process in a regulated manner [5], [24], [25]. In order for Cdks to exhibit enzymatic activity, they must be associated with a binding partner protein called a cyclin. Particular Cdks are associated with one or more cyclins at different points in the cell cycle, and the sequential, temporally coordinated activity of the Cdk/cyclin combinations organizes and orders the molecular events in the cell cycle.

Cdks are obligatory proline-directed serine/threonine kinases. Empirical studies of Cdk substrates indicate a strict requirement for a proline residue one amino acid C-terminal to the acceptor residue (the “+1” site) [12] as well as a strong preference for basic amino acids proximal to the acceptor site, especially arginine or lysine residues at or around the +3 site (*i.e.*, 3 residues C-terminal to the acceptor). These sequence characteristics are supported by X-ray crystal structures that reveal a large binding pocket for docking of the requisite proline residue, and an acidic patch for binding the C-terminal basic region [4], [26], [27]. The identity of other residues surrounding the acceptor site also plays a role, albeit smaller, in Cdk substrate preference. Studies on the catalytic activity of Cdc28 towards *in vitro* peptide phosphorylation show that substrates of different cyclin/Cdk combinations have largely the same primary sequence characteristics, although different combinations do exhibit slightly different preferences [28], [29]. These subtle differences may have a considerable impact on cyclin/Cdk specificity, but other factors such as cyclin abundance, substrate binding and the presence or absence of substrate proteins may also play a significant role.

Studies by Holmes and Solomon [28] directly assayed for amino acid sequence specificity for Cdk phosphorylation. Their approach involved a series of experiments based on a GST fusion constructs, each containing a peptide based on the sequence KSPRK derived from the histone H1 Cdk substrate. The effects of all possible single amino acid substitutions at the −1, +2 and +3 positions (position 0 is the acceptor site, and +1 is the obligatory proline residue) were detailed for *Xenopus laevis* cyclin B-Cdc2 and human cyclin A-Cdc2, cyclin A-Cdk2, cyclin E-Cdk2, and cyclin B-Cdc2. Varying the -1 position was shown to have the least effect, with efficiency of phosphorylation changing, for example, about 2-fold between the worst (Pro) to the best (Gln and Met, followed by His and Gly) amino acids for the *X. laevis* cyclin B-Cdc2. The +2 position shows strong selectivity against Pro, Gln, Glu and Asp, with about one order of magnitude lower reaction efficiency than for Lys, Arg and Met, the amino acids contributing most positively to catalytic efficiency. Nearly all other amino acids are tolerated at this position, showing about 20-60% of wild-type efficiency. The +3 position is the most selective, with Arg and Lys being strongly preferred, His and Pro showing efficiency ∼20% of wild type, and all the others showing efficiency ∼5% of wild type, except for the acidic residues Glu and Asp which showed virtually no activity. The activity profiles for other cyclin-Cdk complexes were essential similar to that for cyclin B-Cdc2.

Our computational strategy focused on two characteristics of Cdk-substrate recognition. We first considered data to determine the primary substrate sequence preference, using published crystal structure and biochemical assay data. Second, we incorporated a number of observations indicating clustering [30] of phosphorylation sites within Cdk substrates. Many of the known Cdk substrates were phosphorylated at multiple sites in their sequence [3], [31]-[34]. Additionally, certain substrates were found to have a specific patch in their structure that bound cyclins (cyclin-binding, or Cy motif) [35], [36], suggesting that the molecular recognition of substrate was influenced by contacts distal from the catalytic site. Biophysical studies on Pho85, a kinase in *S. cerevisiae* homologous to Cdc28, further showed semi-processive phosphorylation—i.e., one kinase-substrate binding event may be followed by several phosphate transfer events without dissociation of the enzyme and substrate proteins [37]. These findings led us to hypothesize that in many cases, Cdk substrates might contain clusters of phosphorylation sites, and therefore that Cdk substrate prediction could be improved not only by optimizing the consensus motif sequence, but also by following consensus site identification with selection of proteins whose sequences are enriched for repeats of that motif. If correct, such an approach will account for the physical mode of phosphorylation and will also overcome the statistical likelihood of false positive predictions based on single site predictions. Multi-site phosphorylation has been previously observed in several different Cdk substrates[37]–[40] One of the best examples of this is phosphorylation of Sic1[39] by Cln-Cdc28 complexes, where multisite phosphorylation acts as a switch that sets a threshold for the onset of DNA synthesis during cell cycle.

## Results

Based on all the preceding considerations, we modeled Cdk substrates by identifying clusters of both the canonical Cdk motif represented by the regular expression [ST]PX[RK] and a PSSM [Table 1] profile generated from Holmes and Solomon's kinetic data [28]. Proteins in the proteome of the budding yeast *Saccharomyces cerevisiae* were scored according to both models, and the distribution of scores for each method was compared to the distribution of scores for sequences from a randomly generated mock proteome (see Methods). Based on these comparisons, we identified a set of candidate Cdk substrate proteins from *S. cerevisiae*, and evaluated that set against experimental data.

**Table 1:** Position-specific scoring matrix representing the Cdk phosphorylation motif

	−1	0	1	2	3
**A**	0.052	0	0	0.049	0.015
**C**	0.046	0	0	0.056	0.015
**D**	0.032	0	0	0.007	0
**E**	0.04	0	0	0.021	0
**F**	0.055	0	0	0.035	0.015
**G**	0.066	0	0	0.021	0.015
**H**	0.052	0	0	0.056	0.029
**I**	0.029	0	0	0.07	0.015
**K**	0.057	0	0	0.15	0.59
**L**	0.052	0	0	0.049	0.015
**M**	0.06	0	0	0.091	0.015
**N**	0.049	0	0	0.021	0.015
**P**	0.02	0	1	0.007	0.029
**Q**	0.075	0	0	0.007	0.015
**R**	0.08	0	0	0.14	0.15
**S**	0.04	0.5	0	0.028	0.015
**T**	0.046	0.5	0	0.063	0.015
**V**	0.04	0	0	0.056	0.015
**W**	0.057	0	0	0.028	0.015
**Y**	0.052	0	0	0.035	0.015

### Clustered canonical motif–based modeling of Cdk substrates

The canonical Cdk phosphorylation motif, represented by the regular expression **[ST]PX[RK]**, represents the most salient features of Cdk phosphorylation site composition and the largest contributions to catalytic efficiency of phosphorylation. It is a highly restrictive statement of a potential Cdk phosphorylation site, in the sense that it does not allow at all for phosphorylation site sequences that may deviate from these features. It accentuates the most influential aspects of site recognition and disregards the rest. The benefit of such an exclusive statement of the phosphorylation motif, then, is that it highlights the most likely phosphorylation sites. However, it is probable that this type of statement will result in underprediction, since not all substrates will have all of their actual phosphorylation sites in these stringent motifs.

**Figure 1:**
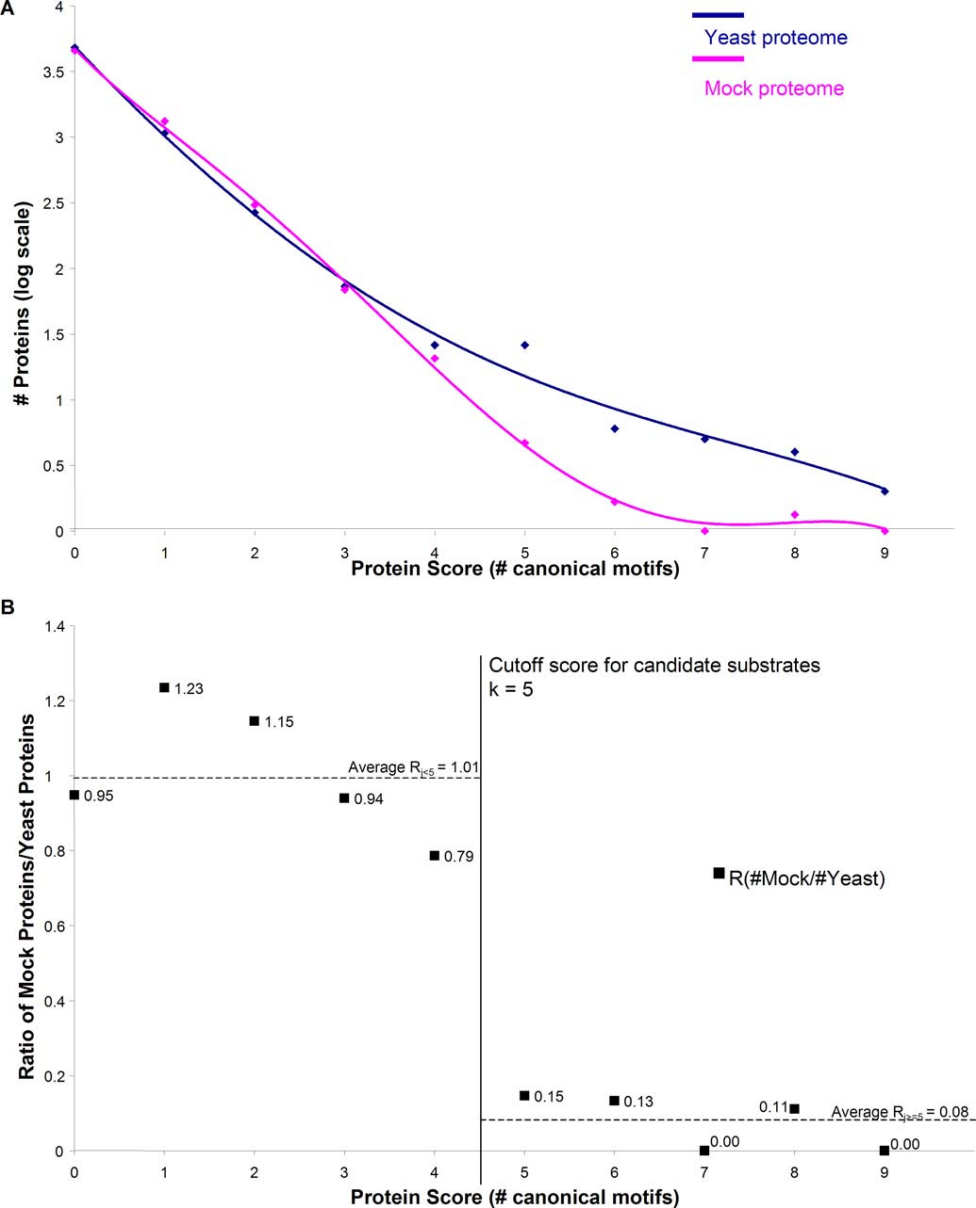
Analysis of Canonical Cdk Motif Clustering in Yeast and Mock Proteomes. (A) The number of proteins having a given score decreases as that score increases. Yeast proteins are represented in navy, and mock proteins are represented in magenta. At low score, (i.e. less than ∼4), yeast and mock are similar– the ratio of mock to yeast, shown by black squares, approximates unity (B). However at higher scores (i.e. 5 and above), yeast proteome contains substantially more proteins than mock (A), and the ratio of mock/yeast approaches zero (B). All proteins from the yeast proteome scoring 5 or higher are considered candidate substrates.

Proteins from the yeast proteome were observed to contain between zero and 9 copies of the canonical phosphorylation motif. The majority of proteins in the yeast proteome (i.e., 4800) had no occurrences of the motif (score = 0), and as a trend, the number of proteins decreased as the score (i.e., the number of canonical motifs) increased [Figure 1A]. A similar general trend was observed in the mock proteome. However, the rate of decrease was higher for the mock than for the yeast proteome [Figure 1A]. In other words, the yeast proteome was enriched for high scoring proteins—suggesting that high scores may indeed be indicative of selection for function as Cdk substrates.

The following procedure was used to predict potential Cdk substrates in an unbiased fashion. For each integral score j between 0 and 9 inclusive, we calculated the ratio r_j_ that represents the ratio of the proportion of proteins from the randomly generated mock proteome with score j to the proportion of yeast proteins with score j [Figure 1B]. It appeared that at low scores of j, r_j_ values were clustered close to unity (i.e., similar in real and mock), but at high scores of j, r_j_ tended toward zero (i.e., enriched in real proteins and therefore candidate Cdk substrate) [Figure 1B]. We determined a cut-off score k that would divide the yeast proteome into 2 groups, a group scoring below k where the number of real proteins is similar to the number of mock proteins, and a group scoring above k, that is enriched for real proteins. Therefore we solved for the value k that minimized the sum of the standard errors of the mean (SEM) over (i) all r_j_ such that j<k, and (ii) all r_j _such that j> = k. We found this value of k to be equal to 5, yielding a lower scoring cluster with an SEM of 0.079 and a higher scoring cluster with an SEM of 0.032. Moreover, this value of k also maximizes the differences between the means of r_j _for the two clusters. The mean of r_j<5_ = 1.01, and the mean of r_j> = 5_ = 0.078.

A total of 38 yeast proteins scored above the threshold value (k = 5) that separated random from significant predicted substrates [Table 2, Table S1]. These 38 included the known Cdk substrates Ace2, Cdc6, Cdh1, Orc2, Sld2, Stb1 and Ste20 [Table 2].[32], [39]–[46] When compared to the results of a proteomic survey of in vitro Cdc28 phosphorylation by Ubersax et al.[47], 25 of the 38 proteins were found in their set of 186 best candidate Cdc28 substrates [Table 2]. In addition, six of the 38 proteins, Cdh1, Lte1, Bem3, Bud3, Ace2 and Ypl267 have been found to physically interact with cyclin/Cdc28 complexes via co-immunoaffinity purification [Table 2] [48].

**Table 2:** Bioinformatic screen for candidates Cdc28 substrates

Protein Name	Candidate (Reg Expr)	Candidate (PSSM)	Borderline (PSSM)	In Vivo^a^ Substrate	In Vitro^b^ Substrate	Cyclin^c^ Interactor
Rad9	9	8.14			x	
Lte1	8	7.48			x	Clb2
Swi5*	8	7.97		Yes	x	
Yer041w	8	5.61				
Ace2*	7	7.14		Yes	x	Clb3
Ase1	7	5.53			x	
Ash1	7	6.41			x	
Sli15	7	6.86				
Bud4	6	5.22			x	
Cdh1	6	4.42		Yes	x	Cln2/Clb3
Fir1	6	5.91			x	
Orc2*	6	6.08		Yes	x	Clb5**
Zrg8	6	5.47				
Bck1	5	4.85			x	
Bem3	5	6.52			x	Cln2
Boi1	5		4.30			
Bud3	5		4.03		x	Clb2
Caf120	5	5.86			x	
Cdc15	5		3.43			
Cdc6	5		3.96	Yes		Clb2
Exo84	5		3.86		x	
Fin1	5				x	
Hcm1	5		3.80		x	
Hpr5	5	4.78			x	
Lre1	5	4.62			x	
Mcm3*	5	4.46			x	
Mse1	5		3.93			
Pak1	5	4.89			x	
Pkc1	5	5.70			x	
Pms1	5	5.31				
Rga2	5	4.86			x	
Sfi1	5				x	
Sir4	5	5.24			x	
Sld2	5	5.69		Yes		
Smc4	5		3.32		x	
Stb1	5	4.75		Yes	x	
Ste20	5		4.33	Yes		Cln2
Ypl267w	5	4.47			x	Cln2
Bni4		5.86				
Iqg1		4.62				
Orc6*		4.44		Yes	x	Clb5**
Plm2		4.96				
Rpo21		4.45				
Ssn2		5.12				
Yjl051w		4.88				
Ymr124w		4.67				
Acc1			3.30			
Bni1			4.36		x	
Chd1			3.86			
Dal81			3.27		x	
Dna2			3.45		x	
Far1			3.46	Yes	x	Cln2/Clb5
Fun30			3.67		x	
Fun31			3.32			
Gac1			3.58			
Hpc2			3.50			
Inp52			3.75			
Kel1			3.36		x	Clb2
Leu1			3.45			
Mds3			4.06			Clb3
Mlp1			3.39		x	
Mps2			3.32			
Mpt1			3.52			
Msb1			3.34		x	
Myo3			3.28		x	
Ndd1			3.46	Yes	x	
Net1			3.38	Yes	x	
Nup60			3.50			x
Pds1			3.28	Yes	x	
Pkh2			3.59			
Rim15			3.38			
Sac3			3.36			
Spa2			3.61		x	
Swi4			3.29			
Tfg1			3.63			
Tra1			4.29			
Tus1			3.39		x	
Ubp2			3.70			
Ulp2			3.25		x	
Ycr033w			4.19			
Ydl239c			3.38			
Ygr271w			3.50			
Yhr080c			3.24			
Yil112w			3.81			
Yjl084w			3.43		x	
Ynr047w			3.45			
Yor066w			4.22		x	
Yor129c			3.32			
Yor177c			3.34			
Yox1			3.29			
Zip1			3.37		x	

a see Supplementary Table S2.

b Reference [47]

c Reference[48]

* Phosphorylation confirmed via mass spectrometry

This method did not predict all known *in vivo* Cdc28 substrates [Supplementary Table S2 reviews and references known Cdc28 substrates]. For example, some known substrates such as Sic1[31], [39], [49], although containing clustered minimal Cdk motifs, do not contain sufficient copies of the full canonical consensus motif to exceed the cut-off value of k = 5.

### Clustered kinetics-based PSSM modeling of Cdk substrates

**Figure 2:**
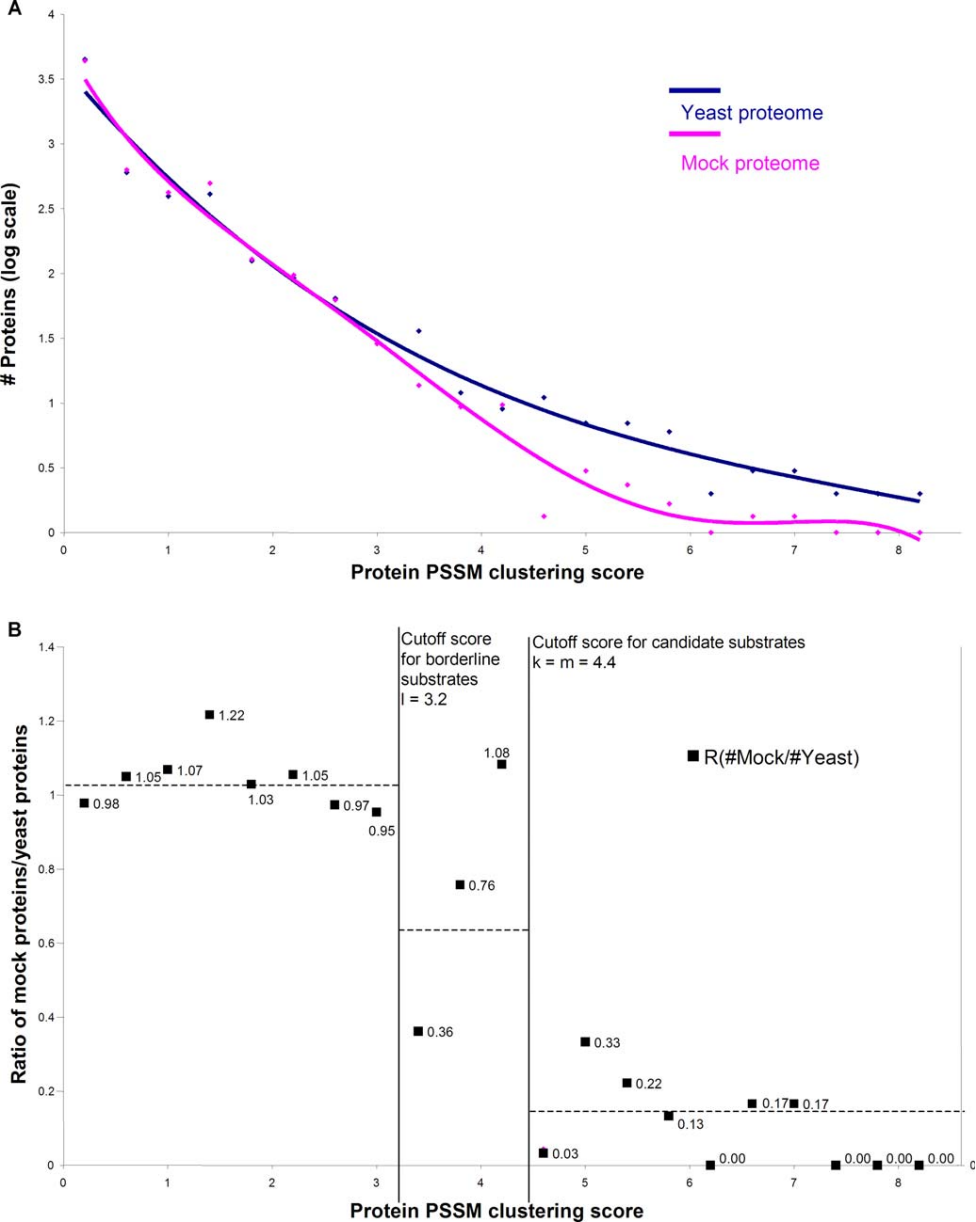
Analysis of kinetic-derived PSSM motif clustering. The number of proteins having a given score decreases as that score increases. Yeast proteins are represented in navy, and mock proteins are represented in magenta. At low score, (I.e. less than ∼3.2), yeast and mock are similar– the ratio of mock to yeast, shown by black squares, approximates unity. (Proteins are grouped in bins 0.4 units wide) However at higher scores (I.e. 4.4 and above), yeast proteome contains substantially more proteins than mock, and the ratio of mock/yeast approaches zero. All proteins from the yeast proteome scoring 4.4or higher are considered candidate substrates. The region between 3.2 and 4.4 is considered a transition region and yeast proteins with these scores are considered borderline candidate substrates

Based on kinetic phosphorylation data [28], we used the PSSM-based approach to model the probability for each of the 20 amino acids at positions –1 through +4 to be present surrounding minimal Cdk phosphorylation motifs [50]. The score for a protein equals the sum of the PSSM score for each potential Cdk site, as defined by Equations 1 and 2 and the PSSM in Table 1. The general trends using this scoring model were similar to those using the canonical consensus regular expression motif scoring system: as the score increased, the occurrence of proteins decreased, with more real proteins than mock proteins at high scores [Figure 2A]. The range of PSSM scores are continuous values, rather than the discrete integral values obtained from regular expression scoring. Therefore, in order to perform analogous discrete analysis for the two scoring systems, we grouped the proteins into bins 0.4 units wide according to their summed PSSM scores.

In this way, we determined that a value of k = 4.4 minimized the sum of the SEM of r_j<k_ and the SEM of r_j> = k_ and maximized the differences between the means of the two clusters; the mean for the lower scoring group r_j<k_ = 0.96 and the mean for the higher scoring group r_j> = k_ = 0.11 [Figure 2B]. Here, there appears to be a region of transition from high to low, between the scores of 3.2 to 4.0 (as opposed to the sharp break between scores of 4 and 5 observed with the regular expression scoring system). To determine the transition area in an unbiased manner, we calculated two values, l and m (such that l< = m) that also minimizes the sum of the SEM of r_j<l_ and the SEM of r_j> = m_. We found values of l = 3.2 and m = 4.4. The values of l and m define respectively the upper boundary of a SEM-minimized cluster of low scoring proteins (with a mean r_j<l = _1.04) where the enrichment of Cdk substrates is likely low, and the lower boundary of a SEM-minimized cluster of high scoring proteins (with a mean r_j> = m = _0.11), which is likely highly enriched for *bona fide* substrates. The region between l and m defines a borderline area that likely contains a mix of substrates and non-substrates. In this case, we found that m = k, defining exactly the same high scoring region likely to contain Cdk substrates, regardless of whether or not we choose to define the borderline region.

The above-described unbiased analysis determined a set of 35 likely Cdk substrate proteins scoring above 4.4, and 55 borderline proteins scoring between 3.2 and 4.4 [Table 2]. Twenty-three of the 35 high scoring candidate substrates were also predicted using regular expression scoring; 6 of the 55 borderline candidates overlapped with regular expression motif scoring candidates [Table 2]. Ace2, Cdh1, Orc2, Sld2, Stb1 were among the known substrates that were predicted both using the canonical regular expression motif and the kinetic PSSM [32], [43]–[45], [51], [52]. Cdc6 [42], [43] and Ste20 [40], [46] are known substrates predicted using the canonical regular expression motif and considered borderline proteins using the kinetic PSSM. Orc6 [43] and Swi5 [34] are known substrates that were predicted by the PSSM method only. Far1, Ndd1, Net 1 and Pds1 are known substrates that were missed using the canonical regular expression motif and considered borderline proteins using the kinetic PSSM [7], [8], [33], [53], [54].

Additionally, 22 of these 35 candidates matched the top scoring *in vitro* substrates [47]. Four candidates, Ace2, Cdh1, Bem3 and Ypl267, were found to physically interact with cyclin/Cdc28 complexes by co-immunoaffinity purification [48]. Twenty-two of the 55 borderline candidates were found to be substrates in the *in vitro* study and two, Bud3 and Far1, were found previously to interact physically with cyclin/Cdc28 complexes.

Using mass spectrometry [38], we were able to determine phosphorylation at Cdk motifs for several predicted substrates. In these experiments, we found *in vitro* phosphorylation of recombinant Mcm3 which had been incubated with ATP and affinity-purified Cdc28 complexes. We also found *in vivo* phosphorylation at Cdk motifs on Ace2, Swi5, Orc2 and Orc6.

## Discussion

We have presented here a model for cyclin-dependent kinase substrates. The model first defines a bioinformatic representation of the Cdk phosphorylation motif, either as a regular expression or a PSSM. In addition, the model proposes that a significant proportion of Cdk phosphorylation occurs on proteins that contain multiple phosphorylation sites. The non-random clustering of potential Cdk sites in particular proteins serves as evidence of biological function selected for by nature.

The canonical motif and PSSM strategies, combined, define a set of 91 candidate Cdk substrate proteins comprising 1.5% the yeast proteome. Of these, 46 (0.73% of the yeast proteome) were defined as strong candidates, either being detected using the canonical-motif scoring method, or scoring above the upper cutoff using PSSM-motif method. Twenty-seven were detected using only the canonical-motif method, 8 using only the PSSM-motif method, and 11 by both methods. The remaining 45 (0.72% of the yeast proteome) predicted candidates were “borderline” PSSM candidates only.

By comparison, only 0.10% of the sequences in the randomized mock proteome scored above the threshold for inclusion as strong candidates, and 0.45% of the sequences in the mock proteome met the score criteria for borderline, PSSM candidates (but not strong candidates). The ratio of candidate substrates detected in yeast-to-candidates substrates detected in mock yields an estimated false positive rate of 14% for the strong candidates and 63% for the borderline candidates. These values indicate that there is indeed clustering on the sequence level beyond what would be expected by random. From them we can infer that ∼40 of the 46 strong candidates and ∼17 of the 45 borderline candidates are *bona fide* Cdk substrates. Thus, although the false positive rate for the borderline candidates is high, that subset is nevertheless not inconsequential to biological researchers, since greater than 1 in 3 are likely to be *bona fide* substrates.

Out of the total set of 91 candidate substrates, 13 proteins (14%) are contained in the set of experimentally characterized *in vivo* substrates. To our knowledge, at the time of writing there are 26 proteins in that set (Table S2); thus 50% of the currently known substrates were detected as candidates. For reasons detailed below, we expect this method to be less than comprehensive, but rather to yield a set of likely candidate substrates useful for biological researchers while maintaining a reasonably low false positive rate. Extrapolating from our false positive and false negative rates, we expect there to be approximately 114 total proteins (1.9% of the yeast proteome) that are Cdc28 substrates.

Many of our candidate substrates were also predicted to contain Cdk phosphorylation sites using other leading phosphorylation detection algorithms, such as Scansite and NetPhosK. Scansite, using a threshold setting of “high” returns 265 yeast proteins (4.2% of the proteome) as candidate Cdk substrates. Of these, 35 are contained in our set of 91 candidate substrates (38%). Scansite predicts 8 of the 24 well-characterized candidate substrates (33%), as compared to the 50% hit rate using our method. When Scansite was run on our random sequence database, 2.8% of the sequences were detected as candidate Cdk substrates -a false positive rate of 67% for Scansite, for Cdk substrate prediction in this dataset. Therefore, although the present method was only somewhat more comprehensive (50% to 33%) than Scansite with respect to true positive detection, it was much more accurate in terms of false positive rate. Our method generates a set of strong candidates with an estimated false positive rate of 14%, while Scansite, even set to high stringency yields a false positive rate of 67%. Scansite yields a false positive rate similar to that of the borderline candidates (63%) generated using the current method.

NetPhosK[9] detected 88 of our 91 (97%) candidates as containing Cdk substrates, using a scoring threshold of 0.60− a similar true positive rate as Scansite. However, our simulations indicate that fully 21% of the proteome, or 1300 proteins, is predicted by NetPhosK to be Cdk substrates, and so the false positive rate is expected to be even higher for NetPhosK than for Scansite. Thus, the major difference between two leading current phosphorylation prediction methods and the one presented here—protein-level motif clustering—is recognized as an increase in accuracy as measured by a reduced false positive rate.

Our method predicts approximately half of the known yeast Cdk substrates. Therefore, in this study, we make no claim at completeness. Instead, we show the utility of a targeted bioinformatic tool that produces a set of predictions that can be validated using experimental techniques. Our pilot proteomic study, in which we assayed for *in vivo* phosphorylation using hypothesis-driven mass spectrometry [38], [55], confirms a number of our predictions [Table 2]. In addition, our predictions are also consistent with many of the high scoring proteins from the high-throughput *in vitro* phosphorylation study by Ubersax *et al.* [47], although most of these are as of yet unconfirmed *in vivo*.

Our model, as it stands, is particularly useful for organisms with small proteomes, such as *S. cerevisiae*. Larger proteomes may be problematic because the false positive rate likely will increase with the number and size of proteins. To extend this procedure effectively may require additional filtering procedures. For example, phosphorylation sites are largely expected to occur on solvent-accessible portions of proteins, particularly loops, so an additional weight could be added to motifs that are expected to occur in such regions, as determined by existing secondary structure prediction [56] or homology modeling algorithms [57]. Incorporating the conservation of phosphorylation motifs across related species into the model might also increase its specificity by adding additional biological restraints. However, this has proven to be not a straightforward task, complicated by the fact that orthologous candidate substrates show homologous regions that are enriched for Cdk motifs, but where in many cases the number and precise positioning of the motifs are *not* very precisely conserved. Supplemental Table S3 shows some examples of the imperfect conservation of Cdk motifs across taxa in Cdk substrates. New algorithms are needed in order to properly account for these factors when performing multiple alignments of Cdk substrates.

Furthermore, the semi-processive physical model [37] of Cdk phosphorylation also suggests that the clustering of sites likely occurs on contiguous surfaces or individual domains of proteins. The average spacing between motifs for candidate substrates identified in our study by canonical motif scoring is 103+/−63 (mean+/−standard deviation) amino acids residues, and by PSSM scoring is 69+/−46 residues. Among the candidate substrates, the subset that overlaps with known, experimentally characterized Cdk substrates, the average spacing was smaller than (63+/−37 for canonical motif scoring, and 38+/−20 for PSSM scoring) but statistically indistinguishable from spacing for the overall set of candidate substrates. Such large spaces between sites suggest that three-dimensional, domain level proximity, rather than simply linear spacing plays an important role in the processivity of Cdk2. Further exploration is necessary to determine the feasibility of using spacing data, or 3-D data for increasing the selectivity of the procedure.

The algorithm missed certain known yeast substrates such as Cdc23 [58] that are thought to contain single phosphorylation sites. However Cdc23 is present in cells in complex with the proteins Cdc16 and Cdc27 [58], both of which also have multiple putative Cdk phosphorylation sties. Therefore, it is reasonable to hypothesize that the kinase recognizes and phosphorylates a surface of the entire complex that is formed by the junction of all three proteins. As data on protein complexes [59]–[61] becomes more comprehensive and reliable, it may become feasible to statistically analyze the presence of Cdk motifs within complexes in a similar manner to that done for individual proteins. We note here that the domain-level clustering of motifs here likely differs from the local clustering observed in the substrates of kinases such as the casein kinases[62]–[64], GSK3[64], [65] and SR specific protein kinases[66], [67], where multiple phosphorylation sites are observed within a single extended motif or repeat region.

The success of the computational procedure presented here stresses the importance of not being limited to local sequence characteristics for functional prediction. The difficulty in the prediction of post-translational modifications and in phosphorylation prediction in particular, is that short, local sequences—even those that match an extremely well defined consensus—can occur frequently by random sequence drift. In the present study, we found useful the fact that Cdk substrates not only have consensus motifs that have been well studied and could be quite precisely defined, but also had the characteristic of site clustering. We incorporated both global and local sequence characteristics of Cdk substrates into a bioinformatic model that proved successful in predicting a significant number of putative substrates. A substantial amount of experimental information obtained by us and other leads us to believe that this set of putative substrates is, in fact, highly enriched for *bona fide* Cdk substrates. This set of proteins includes a substantial proportion of known substrates from previous *in vivo* and *in vitro* studies, as well as substrates that were confirmed as *in vivo* phosphorylation sites by mass spectrometry. In the future, these types of approaches—incorporating biochemical details into bioinformatics, and interfacing bioinformatics with experimental testing—should prove to be a useful strategy in predictive computational biology.

## Materials and Methods

For regular expression consensus motif searches, an algorithm was implemented that scored all proteins in the yeast proteome according to the number of occurrences of the motif. Proteins were scored as the number of phosphorylation motifs within their sequence. For PSSM consensus motif scoring, a PSSM was constructed by assigning a score to each amino acid in each relevant position directly proportional to its effect on catalytic efficiency based on Holmes and Solomon's [28] kinetic data. The specific structure and values of this PSSM can be found in Table 1. These scores were stored in a table—the positions relative to the phosphate acceptor residue was represented on one axis of the table, and the twenty individual amino acids were represented on the other axis. Each protein was scored as follows. First, the information content for each position was calculated from the PSSM using the standard relative entropy definition at each position using the equation:
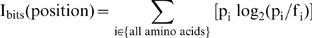
(1)where p_i_ is the observed probability of amino acid i (at a given position) in the motif, and f_i_ is the background frequency of amino acid i in the proteome. The information content at each position should be directly related to its discriminatory power in predicting phosphorylation substrates of Cdk. Then, for each protein, all Ser-Pro and Thr-Pro (the minimal requirement for phosphorylation by Cdk) sequences in a protein sequence were located, and each Ser-Pro and Thr-Pro sequences were scored based on the 5 amino acid window around it (from −1 to +3) around it as:
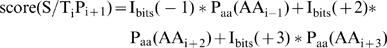
(2)where I_bits_(n) is the total information content at position n, as defined above, and P_aa_(n_k_) is the probability of amino acid n (any one of the 20 amino acids) at position k. This scheme yields a score for each motif that is weighted both by the information content at each position, and by the relative likelihood of the amino acid found at that position. This gives proportionally more weight to positions that possess more discriminatory power.

Proteins are scored as the aggregate of the score of their individual potential Cdk phosphorylation sites. This type of scoring accounts for both the ‘goodness’ of each potential phosphorylation site and the enrichment (and possible clustering) of potential sites within the protein sequence. The score of a protein is defined as the sum of all scores (S/T_i_P_i+1_) for that protein sequence. The scorings of protein using the regular expression version of the phosphorylation motif can also be represented using this system, by assigning a value of one for relative information to each relevant position and assigning a score of 1 for each match to the regular expression found in a given protein sequence.

A set of randomly generated amino acid sequences, collectively having identical amino acid composition and protein length distributions as the actual yeast proteome, was used as a negative control. The Cdk phosphorylation motifs found in this ‘mock proteome’ represent the amino acid distribution if it were truly random. Deviations from the random distribution are likely to result from selective pressure on protein sequences, and therefore to reflect biological functionality as phosphorylation substrates.

Scripts were written in PERL on and executed on a multi-CPU Sun server running Solaris 10 to find putative phosphorylation sites and compute their scores for each yeast protein sequence and mock protein sequence using the formulae (1) and (2) as described above. Multiple alignments were performed using the World-Wide Web based clustalW [68], [69] server hosted by EMBL.

## Supporting Information

Table S1 Accession numbers and descriptions of candidate substrates.
(0.03 MB DOC)

Table S2 Compilation of currently known substrates of Cdc28.
(0.04 MB DOC)

Table S3 Conservation and alignment of Cdk phosphorylation motifs. Sequences matching canonical and minimal Cdk motifs are highlighted in bold, demonstrating imperfect conservation of motifs across organisms. While some motifs show near perfect alignment, other sites appear in the same general area across the organisms, but are not aligned precisely by the ClustalW organism, either due to differing numbers of sites, or different locations within the protein sequence. Such imperfect alignment corroborates the proposition that selection has occurred on Cdk substrates to favor domain-level clustered phosphorylation. Note for example, that the S. cerevisiae Orc6 (example A) sequence contains four motifs around residue 105–124, three of which nearly perfectly align with the corresponding A. gossypii sequence, while K. lactis contains only two corresponding motifs, and C. albicans only one. Another good example is in the region corresponding to residues 300–340 in S. cerevisiae Swi5 (example C), which contains four Cdk motifs. The corresponding region in A gosyppi contains 5 motifs, and in C. albicans contains 6 motifs, none of which align well with the S. cerevisiae motifs, while the K. lactis contains only 1 single motif in the regions.
(0.04 MB DOC)
