# Supplementary material for: Prediction of Cyclin-Dependent Kinase Phosphorylation Substrates
Source: PLoS One. 2007 Aug 1;2(8):e656. doi: 10.1371/journal.pone.0000656 (PMC1924601; doi:10.1371/journal.pone.0000656)
Supplement: Table S1 — Accession numbers and descriptions of candidate substrates. (0.03 MB DOC) [file pone.0000656.s001.doc]

# SUPPLEMENTARY TABLES

# Table S1: Acession numbers and descriptions of candidate substrates

# A: Candidate substrates using canonical regular expression method

>gi|6319295|ref|NP_009378.1| putative GTP-exchange protein; Lte1p

>gi|6319328|ref|NP_009411.1| protein kinase domain; Cdc15p

>gi|6319363|ref|NP_009445.1| Protein Kinase C; Pkc1p

>gi|6319386|ref|NP_009468.1| BEM1-binding protein; Boi1p

>gi|6319534|ref|NP_009616.1| origin recognition complex subunit 2; Orc2p

>gi|6319578|ref|NP_009660.1| Exo84p

>gi|6319632|ref|NP_009714.1| Sli15p

>gi|10383756|ref|NP_009879.2| involved in laminarase resistance; Lre1p

>gi|10383775|ref|NP_009914.2| involved in bud site selection; Bud3p

>gi|10383801|ref|NP_009991.2| Transcription factor (fork head domain); Hcm1p

>gi|6320335|ref|NP_010415.1| Cell cycle-dependent filament between nuclei; Fin1p

>gi|6320423|ref|NP_010503.1| cell cycle arrest protein; Rad9p

>gi|6320433|ref|NP_010513.1| regulator of silencing at HML, HMR, and telomeres; Sir4p

>gi|6320587|ref|NP_010667.1| Contains a Rho-GAP domain and two LIM domains. Has strong similarity to Rga1p. Has some similarity to all known Rho-GAPs.; Rga2p

>gi|6320803|ref|NP_010882.1| component of DNA replication initiator complex; Mcm3p

>gi|6320870|ref|NP_010949.1| Putative participant in 3' mRNA processing; Fir1p

>gi|6320871|ref|NP_010950.1| Zrg8p

>gi|6320880|ref|NP_010959.1| Yer041wp

>gi|6320976|ref|NP_011055.1| DNA polymerase alpha suppressing protein kinase; Pak1p

>gi|6321435|ref|NP_011512.1| protein required for Clb2 and Ase1 degradation; Cdh1p

>gi|6321780|ref|NP_011856.1| serine/threonine protein kinase; Ste20p

>gi|6322266|ref|NP_012341.1| pre-initiation complex component; Cdc6p

>gi|6322366|ref|NP_012440.1| MEKK serine/threonine kinase; Bck1p

>gi|6322369|ref|NP_012443.1| DNA helicase; Hpr5p

>gi|6322551|ref|NP_012625.1| involved in bud site selection; Bud4p

>gi|6322663|ref|NP_012736.1| probable purine nucleotide-binding protein; Ash1p

>gi|6322741|ref|NP_012814.1| Sld2p

>gi|6323026|ref|NP_013098.1| Sfi1p

>gi|6323115|ref|NP_013187.1| SMC chromosomal ATPase family member; Smc4p

>gi|6323160|ref|NP_013232.1| zinc finger transcription factor; Ace2p

>gi|6324020|ref|NP_014090.1| Interacts with the putative transcription factor Sin3p; Stb1p

>gi|6324051|ref|NP_014121.1| Caf120p

>gi|6324247|ref|NP_014317.1| MutL homolog, similar to Mlh1p, associates with Mlh1p, possibly forming a heterodimer, Pms1p and Msh1p act in concert to bind to a Msh2p-heteroduplex complex containing a G-T mismatch; Pms1p

>gi|6324540|ref|NP_014609.1| Mitochondrial glutamyl-tRNA synthetase; Mse1p

>gi|6324632|ref|NP_014701.1| encodes component of the spindle midzone; Ase1p

>gi|6324988|ref|NP_015056.1| Ypl267wp

>gi|6325142|ref|NP_015210.1| Bem3p

# B: Candidate substrates using kinetic-based PSSM method

*Strong Candidates (4.4 or higher)*

>gi|6319295|ref|NP_009378.1| putative GTP-exchange protein; Lte1p 7.48642984279483

>gi|6319363|ref|NP_009445.1| Protein Kinase C; Pkc1p 5.69686717591301

>gi|6319534|ref|NP_009616.1| origin recognition complex subunit 2; Orc2p 6.07827596024909

>gi|6319632|ref|NP_009714.1| Sli15p 6.86222241510009

>gi|10383756|ref|NP_009879.2| involved in laminarase resistance; Lre1p 4.62191434142943

>gi|6320061|ref|NP_010141.1| RNA polymerase II large subunit; Rpo21p 4.4456869042551

>gi|6320350|ref|NP_010430.1| transcriptional activator; Swi5p 7.9698327990919

>gi|6320423|ref|NP_010503.1| cell cycle arrest protein; Rad9p 8.14472633087621

>gi|6320433|ref|NP_010513.1| regulator of silencing at HML, HMR, and telomeres; Sir4p 5.23823620976599

>gi|6320587|ref|NP_010667.1| Contains a Rho-GAP domain and two LIM domains. Has strong similarity to Rga1p. Has some similarity to all known Rho-GAPs.; Rga2p 4.85603491122444

>gi|6320651|ref|NP_010731.1| transcription factor; Ssn2p 5.12268674882761

>gi|6320709|ref|NP_010789.1| Plm2p 4.95975740058792

>gi|6320803|ref|NP_010882.1| component of DNA replication initiator complex; Mcm3p 4.46408609393804

>gi|6320870|ref|NP_010949.1| Putative participant in 3' mRNA processing; Fir1p 5.91452585665664

>gi|6320871|ref|NP_010950.1| Zrg8p 5.47288491683077

>gi|6320880|ref|NP_010959.1| Yer041wp 5.61929496039957

>gi|6320976|ref|NP_011055.1| DNA polymerase alpha suppressing protein kinase; Pak1p 4.89523697102194

>gi|6321435|ref|NP_011512.1| protein required for Clb2 and Ase1 degradation; Cdh1p 4.42399669943043

>gi|6321910|ref|NP_011986.1| 50-kDa subunit of ORC; Orc6p 4.44212508521847

>gi|6322366|ref|NP_012440.1| MEKK serine/threonine kinase; Bck1p 4.8466447864662

>gi|6322369|ref|NP_012443.1| DNA helicase; Hpr5p 4.78379354035651

>gi|6322410|ref|NP_012484.1| Yjl051wp 4.88315174773827

>gi|6322551|ref|NP_012625.1| involved in bud site selection; Bud4p 5.22379565619648

>gi|6322663|ref|NP_012736.1| probable purine nucleotide-binding protein; Ash1p 6.4092968282088

>gi|6322741|ref|NP_012814.1| Sld2p 5.69205600652809

>gi|6323160|ref|NP_013232.1| zinc finger transcription factor; Ace2p 7.13630314431875

>gi|6323772|ref|NP_013843.1| Ymr124wp 4.66609033700924

>gi|6324020|ref|NP_014090.1| Interacts with the putative transcription factor Sin3p; Stb1p 4.75303870850037

>gi|6324051|ref|NP_014121.1| Caf120p 5.86352938820474

>gi|6324096|ref|NP_014166.1| Is required to link Chs3p and Chs4p to the septins; Bni4p 5.29725469961202

>gi|6324247|ref|NP_014317.1| MutL homolog, similar to Mlh1p, associates with Mlh1p, possibly forming a heterodimer, Pms1p and Msh1p act in concert to bind to a Msh2p-heteroduplex complex containing a G-T mismatch; Pms1p 5.31213856874529

>gi|6324632|ref|NP_014701.1| encodes component of the spindle midzone; Ase1p 5.5350692268525

>gi|6324988|ref|NP_015056.1| Ypl267wp 4.46709734306126

>gi|6325014|ref|NP_015082.1| Similar to mammalian IQGAP proteins; Iqg1p 4.61970159840648

>gi|6325142|ref|NP_015210.1| Bem3p 6.51768507964256

*Borderline Candidates (3.2 to 4.4)*

>gi|6319300|ref|NP_009383.1| SNF2 protein family; Fun30p 3.67386968606933

>gi|6319302|ref|NP_009385.1| Serine/threonine kinase; Fun31p 3.32779715907709

>gi|6319318|ref|NP_009401.1| nuclear pore protein; Nup60p 3.49737072828042

>gi|6319328|ref|NP_009411.1| protein kinase domain; Cdc15p 3.43434288032492

>gi|6319386|ref|NP_009468.1| BEM1-binding protein; Boi1p 4.30491803243493

>gi|6319578|ref|NP_009660.1| Exo84p 3.86262681387518

>gi|6319692|ref|NP_009774.1| highly charged, basic protein; Hpc2p 3.50310166196382

>gi|10383775|ref|NP_009914.2| involved in bud site selection; Bud3p 4.03354672231355

>gi|10383795|ref|NP_009962.2| Ycr033wp 4.18587289675287

>gi|10383801|ref|NP_009991.2| Transcription factor (fork head domain); Hcm1p 3.79866509950851

>gi|6319962|ref|NP_010042.1| Ydl239cp 3.38302378425701

>gi|6320318|ref|NP_010398.1| 42-kDa nuclear protein; Pds1p 3.28208059666941

>gi|6320363|ref|NP_010443.1| Leucine permease transcriptional regulator; Sac3p 3.36214962800651

>gi|6320491|ref|NP_010571.1| synaptonemal complex protein; Zip1p 3.37239734460611

>gi|6322079|ref|NP_012154.1| Yil112wp 3.8062250778668

>gi|6322158|ref|NP_012233.1| Ulp2p 3.2476835810299

>gi|6322214|ref|NP_012289.1| Transcriptional activator for allantoin and GABA catabolic genes, contains a Zn[2]-Cys[6] fungal-type binuclear cluster domain in the N-terminal region; Dal81p 3.2706588709664

>gi|6320957|ref|NP_011036.1| transcription factor; Swi4p 3.28885445271342

>gi|6321012|ref|NP_011091.1| transcriptional regulator; Chd1p 3.86069594020282

>gi|6321076|ref|NP_011154.1| Trehalose-associated protein kinase related to S. pombe cek1+; Rim15p 3.38312195814193

>gi|6321241|ref|NP_011318.1| negative regulator of early meiotic genes; Mds3p 4.0627197458069

>gi|6321363|ref|NP_011440.1| nuclear envelope/ER protein involved in chromosomal segregation; Mps2p 3.32485579263248

>gi|6321429|ref|NP_011506.1| isopropylmalate isomerase; Leu1p 3.45094625491412

>gi|6321625|ref|NP_011702.1| Transcription factor TFIIF large subunit; Tfg1p 3.63259306361153

>gi|6321710|ref|NP_011787.1| Ygr271wp 3.49892733400683

>gi|6321780|ref|NP_011856.1| serine/threonine protein kinase; Ste20p 4.33034118458476

>gi|6321872|ref|NP_011948.1| Yhr080cp 3.24281759947198

>gi|6321891|ref|NP_011967.1| ATM/Mec1/TOR1+2-related; Tra1p 4.28915110215966

>gi|6321952|ref|NP_012028.1| involved in cell fusion and morphology; contains six Kelch repeats; Kel1p 3.35709637914349

>gi|6321958|ref|NP_012034.1| DNA replication helicase; Dna2p 3.45494349590187

>gi|6322266|ref|NP_012341.1| pre-initiation complex component; Cdc6p 3.96186688669668

>gi|6322304|ref|NP_012378.1| Cdc28p kinase inhibitor; Far1p 3.462905548289

>gi|6322377|ref|NP_012451.1| Yjl084cp 3.42700370058516

>gi|6322385|ref|NP_012459.1| Net1p 3.37528486727066

>gi|6322720|ref|NP_012793.1| myosin I; Myo3p 3.28400547844697

>gi|6322948|ref|NP_013021.1| colied-coil protein (putative), similar to myosin and TPR; Mlp1p 3.34860522109366

>gi|6323007|ref|NP_013079.1| spindle pole antigen; Spa2p 3.61150533569448

>gi|6323115|ref|NP_013187.1| SMC chromosomal ATPase family member; Smc4p 3.32284353576142

>gi|6323457|ref|NP_013529.1| 1307 AA, hydrophilic protein.; Tus1p 3.39231869036813

>gi|6323614|ref|NP_013685.1| Yox1p 3.28873940486917

>gi|6323647|ref|NP_013718.1| involved in protein synthesis; Mpt1p 3.52105503090061

>gi|6324058|ref|NP_014128.1| contains formin homology domains; homologous to BNR1 (BNI1 related protein); Bni1p 4.36152439483118

>gi|6324224|ref|NP_014293.1| inositol polyphosphate 5-phosphatase; Inp52p 3.75197294403132

>gi|6324343|ref|NP_014413.1| acetyl-CoA carboxylase; Acc1p 3.30021634274157

>gi|6324375|ref|NP_014445.1| Ynr047wp 3.44565409841604

>gi|6324472|ref|NP_014541.1| Ser/Thr protein kinase; Pkh2p 3.58650249992216

>gi|6324540|ref|NP_014609.1| Mitochondrial glutamyl-tRNA synthetase; Mse1p 3.92538918316993

>gi|6324640|ref|NP_014709.1| Yor066wp 4.21542045485643

>gi|6324698|ref|NP_014767.1| Ubiquitin-specific protease; Ubp2p 3.70253907320063

>gi|6324703|ref|NP_014772.1| Yor129cp 3.32704822318879

>gi|6324751|ref|NP_014820.1| Yor177cp 3.34740979779855

>gi|6324752|ref|NP_014821.1| Regulatory subunit for Glc7p; Gac1p 3.5864422642509

>gi|6324762|ref|NP_014831.1| involved in polarized growth; Msb1p 3.33689550081313

### >gi|6324948|ref|NP_015017.1| Ndd1p 3.45765084488116
