# Supplementary material for: Prediction of Cyclin-Dependent Kinase Phosphorylation Substrates
Source: PLoS One. 2007 Aug 1;2(8):e656. doi: 10.1371/journal.pone.0000656 (PMC1924601; doi:10.1371/journal.pone.0000656)
Supplement: Table S2 — Compilation of currently known substrates of Cdc28. (0.04 MB DOC) [file pone.0000656.s002.doc]

### **Table S2. Compilation of currently known substrates of Cdc28**

**Ace2:** Homolog of Swi5 (see below), mutation of phosphorylation sites leads to change in nuclear localization; *in vivo* phosphorylation characterized in detail as part of this thesis[50]

**Ask1:** Member of the DASH complex that binds to microtubules and kinetochores and regulates their association[69]

**Cdc3:** Member of the septin ring present at bud necks; phosphorylation by Cdc28 at the end of G_1_ promotes efficient septin ring disassembly_[70]_

**Cdc6:** Participant in preparing DNA for replication; interacts with Cdc28 via the N-terminal 47 amino acids; phosphorylation targets Cdc6 for ubiquitin-mediated degradation phosphorylation may decrease affinity to Mcm DNA-licensing proteins; phosphorylation of the human homologue hCdc6 by cyclin A-Cdk2 mediates localization from the nucleus to the cytoplasm[41,42]

**Cdc16-Cdc23-Cdc27:** Components of the anaphase promoting complex (APC); all three are phosphorylated by Cdc28 *in vitro*, mutating Cdc28 consensus site blocks *in vitro* phosphorylation in *in vivo* activity of the APC during mitosis. Cdc28 phosphorylation of the APC triggers the onset of anaphase [58]

**Cdh1 (aka Hct1):** Participates in APC mediated degradation of Ase1 and b-type cyclins; Cdk phosphorylation of Cdh1 blocks interaction between Cdh1 and the APC, inhibiting Cdh1-dependent APC-mediated degradation of b-type cyclins and Ase1[31]

**Cln2:** Phosphorylated *in vivo* in a Cdc28 dependent manner, and degraded in a Cdc28 and Cdc34 ubiquitin ligase dependent manner. It has also been proposed that **Cln3**, a cyclin that drives transcription early in cell cycle is phosphorylated by Cln-Cdc28, possibly autocatalytically [71,72]

**Dpb2:** Subunit of DNA polymerase epsilon complex; Cdc28 phosphorylation during late G_1_ hypothesized to mediate polymerase epsilon function[73]

**Far1:** Inhibitor of Cln1- and Cln2-Cdc28. Phosphorylation of Far1 by Cln-Cdc28 mediates ubiquitination and degradation of Far1, allowing the activation of Cln-Cdc28 activity in a feed-forward fashion [3,74]

**Kar9:** Phosphorylated in a Clb-Cdc28 dependent manner to mediate proper spindle alignment[75]

**Mcm complex:** Complex necessary for licensing origins for replication; phosphorylation by Clb-Cdc28 after

DNA synthesis contributes to prevention of premature re-replication; possible targets include Mcm2 and Mcm3[42,76]

**Mps1:** Phosphorylation of Mps1 by Cdc28 regulates spindle pole body duplication[77]

**Ndd1:** Phosphorylated at sequence “RTPLR” in a Clb-Cdc28 dependent manner; phosphorylation is required for Clb-dependent mitotic gene transcription[52]

**Net1:** Net1 sequester the mitotic phosphatase Cdc14 in the nucleolus; phosphorylation by Clb-Cdc28 underlies the release of Cdc14 at the appropriate time[7,8]

**Orc6-Orc2:** Components in origin recognition complex (ORC) that participates in binding DNA replication licensing factors; phosphorylation

by Clb-Cdc28 contributes to prevention of premature re-replication; shows phosphorylation-dependent gel shift that disappears upon mutation of all Cdc28 consensus sites. [42]

**Table S2, continued**

**Pds1:** Inhibits the onset of anaphase by binding and sequestering the Esp1 protease that cleaves the cohesin complexes that hold sister chromatids together. Binding of Pds1 to Esp1 was reported to depend in Cdc28 phosphorylation[53]

**Sic1:** Inhibitor of Clb-Cdc28. Phosphorylation of Sic1 by Cln-Cdc28 as well as Pcl1-Pho85 cyclin dependent kinase is necessary; phosphorylation leads to ubiquitin-dependent degradation of Sic1, which allows the cell to pass Start. *In vitro* and *in vivo* phosphorylation sites have been mapped using mass spectrometry. [30,78]

**Sld2:** Required for association of Sld2 with its binding partner Dpb11; necessary for DNA replication[43]

**Spc42:** Phosphorylation of Spc42 by Cdc28 regulates spindle pole body duplication[77]

**Stb1:**  Involved in transcription, binds to cell cycle transcription factor Swi6 and is necessary for Start in the absence of Cln3. Phosphorylated by Cln-Cdc28 both *in vitro* and *in vivo.[44]*

**Ste20:** Participates in mating pheromone response pathway. Associates with Cln-Cdc28. Phosphorylation sites have been mapped using mass spectrometry.[39,79]

**Swi5:** Cell cycle related transcription factor. Phosphorylation sequesters Swi5 in cytoplasm; unphosphorylated Swi5 is present in the nucleus.[33]
