# Supplementary material for: Prediction of Cyclin-Dependent Kinase Phosphorylation Substrates
Source: PLoS One. 2007 Aug 1;2(8):e656. doi: 10.1371/journal.pone.0000656 (PMC1924601; doi:10.1371/journal.pone.0000656)
Supplement: Table S3 — Conservation and alignment of Cdk phosphorylation motifs. Sequences matching canonical and minimal Cdk motifs are highlighted in bold, demonstrating imperfect conservation of motifs across organisms. While some motifs show near perfect alignment, other sites appear in the same general area across the organisms, but are not aligned precisely by the ClustalW organism, either due to differing numbers of sites, or different locations within the protein sequence. Such imperfect alignment corroborates the proposition that selection has occurred on Cdk substrates to favor domain-level clustered phosphorylation. Note for example, that the S. cerevisiae Orc6 (example A) sequence contains four motifs around residue 105–124, three of which nearly perfectly align with the corresponding A. gossypii sequence, while K. lactis contains only two corresponding motifs, and C. albicans only one. Another good example is in the region corresponding to residues 300–340 in S. cerevisiae Swi5 (example C), which contains four Cdk motifs. The corresponding region in A gosyppi contains 5 motifs, and in C. albicans contains 6 motifs, none of which align well with the S. cerevisiae motifs, while the K. lactis contains only 1 single motif in the regions. (0.04 MB DOC) [file pone.0000656.s003.doc]

**Table S3. Conservation and alignment of Cdk phosphorylation motifs.** Sequences matching canonical and minimal Cdk motifs are highlighted in bold, demonstrating imperfect conservation of motifs across organisms. While some motifs show near perfect alignment, other sites appear in the same general area across the organisms, but are not aligned precisely by the ClustalW organism, either due to differing numbers of sites, or different locations within the protein sequence. Such imperfect alignment corroborates the proposition that selection has occurred on Cdk substrates to favor domain-level clustered phosphorylation. Note for example, that the *S. cerevisiae* Orc6 (example A) sequence contains four motifs around residue 105 – 124, three of which nearly perfectly align with the corresponding *A. gossypii* sequence, while *K. lactis* contains only two corresponding motifs, and *C. albicans* only one. Another good example is in the region corresponding to residues 300-340 in *S. cerevisiae* Swi5 (example C), which contains four Cdk motifs. The corresponding region in *A gosyppi* contains 5 motifs, and in *C. albicans* contains 6 motifs, none of which align well with the *S. cerevisiae* motifs, while the *K. lactis* contains only 1 single motif in the regions

**A. Multiple sequence alignment between *S. cerevisiae* Orc6 protein and the closest homologue in three related yeasts, *Kluyveromyces lactis (Klac), Ashbya gossypii (Agos)* and *Candida albicans (Calb)*.**

Klac MSTQQVRTCVTDLLGVKNDSN-VDWQDQRLKKVASTTATLYNVSVS-KVMLKNSEELARC 58

Agos MSVSQVRQSVSEILGLNAQEE**TP**DWNSGRLKRMAATTATLYNVSLN-KVMLKQPEEIARC 59

Scer MSMQQVQHCVAEVLRLDPQEK-PDWSSGYLKKLTNATSILYNTSLN-KVMLKQDEEVARC 58

Calb --------MSSSQARKALQDVIPNYLGEF**TP**KLLDYINSLYQLSLRKQAILPNKSEIARF 52

:. :. :: . :: **: *: :.:* : .*:**

Klac HICALIAFQKLAEKYDNDLPYSQEKIPLPPDQVSKLVNIFKRNIWPH**SPQK**DTEGQLLKF 118

Agos HICAYLAAEKLSEKYEPELQYYREKIPLEPRKTVKLVGLFKQTLWTS**SPVR**NLNL**SPSPK** 119

Scer HICAYIASQKMNEKHMPDLCYYIDSIPLEPKKAKHLMNLFRQSLSNS**SPMK**QFAW**TPSPK** 118

Calb HLCAVVIVEKYKQSFELP**TP**DVS-RIPTQPKVAAKLLDTFRELIEQISAAS**TP**VS**SPKK**V 111

*:** : :* :.. ** * . :*:. *:. : *.

Klac DDTA**SP**SVRKSAVKNARFTGIDPKSLQEQLFQ**TPSK**SRTKSGIQPLKNVDL**SPSK**GSRSS 178

Agos **K**DGGRLSARDPVELRAELFG**TPVK**RAGAVTLPGAGEL**SPTK**QL**SPSK**-----PM**SPVK**PS 174

Scer **K**NK-----R**SPVK**NGGRFTSSDPKELRNQLFG**TPTK**VRKSQNNDSFVIPELPPMQTNE**SP** 173

Calb KPP------------------------SQ**SP**S**TPTK**SRTSKENLKSG----------**SP**L 137

. . : .. .

Klac --**VR**RKLVFEADTENGLPLPI------------LA**TP**KNKTDMIN--IPQVFGSVEDDPE 222

Agos --**PR**RRLAFEDEDEDYEPDAS------------PQP**SPRR**SIFPGGFEPDPDESEFD**SPT** 220

Scer SITRRKLAFEEDEDEDEEEPGNDGLSLKSHSNKSITGTRNVDSDEYENHESDPTSEEEPL 233

Calb KRLRAEMLQEDQVNGN**SP**DGQ--------------------------LKDVD**SP**FNPKKR 171

* .: * : : : . .

Klac **SPLR**Q**SPFR**D**SPSK**Y**TPSPRK**--------KRGKYNEWNMLYKKYYRPSTAELVTLCNEFE 274

Agos **KSP**FK**SP**TR**SPTK**SAQS**SPRK**--------KRGEYNQWNMLYKKYYRI**TP**EEIVGLCNQFE 272

Scer GVQESRSGRTKQNKAVGKPQSELKTAKALRKRGRIPNSLLVKKYCKMTTEEIIRLCNDFE 293

Calb KESKAG**TP**THKVYKYDKKHVS---------IADFIAFCNTFLIPGDITAKMVGTFLTHQH 222

. . . . :. : : .. .

Klac LPEEVTATIISEFNNNATYLAYPTQLVCGLVLLCSFVVFNQQRNQDSTIDNKLMKKMAAQ 334

Agos LPSRVAFQVLDCFGMHATYLVYPAQLVCGLVMLCCFVVHHRKRASDPTIDDYLLKKMCAL 332

Scer LPREVAYKIVDEYNINASRLVCPWQLVCGLVLNCTFIVFNERRRKDPRIDHFIVSKMCSL 353

Calb KFLKKSDWSLACGMVYAAYIRINNRLLAQSVGTKSEFTKQLLQYQKGGLSLGAMQSWCGI 282

. : : *: : :*:. * .. : : .. :. :.. ..

Klac MRTTNDEDIMEAIKITKELIDGEKWYRELRVKFDYYDGSDFDNAIAVRIGSMLQDEYEVV 394

Agos LRSNSTSDVLEAMKITKELLDGENWYRALKIEYDYYDAADFEQSLAIRLGSMLQNSNVIA 392

Scer MLTSKVDDVIECVKLVKELIIGEKWFRDLQIRYDDFDGIRYDEIIFRKLGSMLQTTNILV 413

Calb IEEWIQ-DEPWIQEIEKTYAYGSKTAEETRNSFERKAKIGEGWDLMEQFGAMIHGETISL 341

: * :: * *.: . : :: : ::*:*::

Klac SEEQYSNWKRRIMVDLSLRDGT 416

Agos STEQFEEWKKKVMLDLSLRDGS 414

Scer TDDQYNIWKKRIEMDLALTEPL 435

Calb SSHQEEYYKNWRKEALEKCDQL 363

: .* . :*. * :

**B. Multiple sequence alignment between *S. cerevisiae* Sld2 protein and the closest homologue in related yeasts.**

Agos -----------------------------------MSRTSAGELDVLKVQLKTWERQFLE 25

Klac MGNFNNLRVLISFFAFILTTFMNGIKETDTVNAPLLLVISSASMDALKIEIKLWERAFEK 60

Scer --------------------------------------MYSFELDKLKIELKTWEHDFID 22

Calb -----------------------------------------MDIVEIKSKIKEWEYAFRK 19

.: :* ::* ** * .

Agos ENGR**SPIK**EDIKAHPEIRRKYKEYTSLKKLLSKGNAAVTGQKHG**SPSR**ATHP**TPQK**HIDA 85

Klac EHGRLPEKDDIKKDKEVKRKYKQYAQLKKDATVKPSVEVNVEQT-------PVKSHVNNT 113

Scer KNKREPTRDDIKSLRTVRQMYKQYSTLKKKQSLQRQKVDTQESVEL-----PAHKKDHDE 77

Calb QHNKLPSKADIKDDVEIHKLYSLYKSIKSGQQQKPSKQETVNEPASVQSS-PVKRNDY**SP** 78

:: : * : *** ::: *. * :*. : *. : .

Agos EIELGP**TP**QIYGKVVSLFEMHI**SPLK**KVPVRQLDADSSETCI**SP**DLHSQDVAQSELLTDI 145

Klac KAEFGP**TP**QMNGKLVSIFEMQVSAMKTHQSDQEDIVGS------PVRSNDLVR------- 160

Scer VVEIGP**TP**QVYGKAISIFDMNL**SPIK**PIYMTFTNNIDVNNDNSKTISNES**SPRK**TILLKS 137

Calb RGELGP**TP**QANGRVLSIFDLKM**TP**PDS**SP**LKHKSDKAS---------------------- 116

*:***** *: :*:*:::::. . .

Agos S-----------CQAKRQLDFSVT-------PHA**SPVK**AVQPLLLNAPDLRFEAIPHART 187

Klac ----------------RQLNFSIT-------PNS**SPMK**QVPNLSAN--------IVVSRP 189

Scer **SP**ADRTLVAEPISSVKRQLNFQMLNASSTR**TP**TS**SPCK**NRNGKLVEIKKC**SP**TINPPLES 197

Calb -----------------PSAFAMP-------PPQ**SPVK**NIIE**TPTK**SK------NKSFV**T** 146

* : * ** * : .

Agos K-----YGPN**SPVK**FDG---------------DVTLTLSQ**TP**--LQAKLAQASEGY**SPSP** 225

Klac K-----YGPN**SPMR**IG----------------DIGLQLSE**TP**KTLGRTLDLKS**SP**F**SPSP** 228

Scer GKPSGYYGPN**SPLK**LDEENIHLNISLNSSTKRRLQIAYPSLQK**TPSK**DQADISTSF**SPSP** 257

Calb **PIK**GRKIVFE**TP**SYLN-----------------KHRQNPQ**TP**DSHNNNNNNNNNTVINFS 189

::* :. .. . .

Agos LIKRP-AKPLSQLAKEYEDIVEELK----EVDHAAAVRNLGGLLQQEE--------ENTQ 272

Klac LIKRP-VKTLSQLAKEHAIIKDEFESNPEDFSEFTAIRTLMEKLMQEE--------HMDI 279

Scer LIRRPLTKSLIELAREHTEIVKEFGVLQEEDIEEEEEGEEGENGYDEKNHEDDFGLEDEL 317

Calb V**SPSP**-**FK**TQRSIGKRLTEVYNTSLKEAEDLKSFNLEEEFQSHEEQES--------EETE 240

: * *. .:.:. : . : :*. .

Agos EAEAAEPSAT-----------RSDRKRRKNKVRPALVT-LEEEIPQ-------GNLHEQL 313

Klac IEEGDEGTVGNETESNYVRKVKTIKKRTKAKMRPAALNEKSTNIPD-------KNVHEQL 332

Scer IRPKVVKDIFQEDDDNDDSQAREDTFIRKRPKRRKVIRRLRDNDPETETAGFERDVHKEL 377

Calb TTTNNDRKIAP----------RSKRTQKRSTRRVKMAPRPVNSKPSLEN----VNLQDHI 286

: : * . *. :::..:

Agos VKLRQKALDKFNGNDPNDSSDEEKKT---------SATSAKPAKARKRKYNTVSDNFRRL 364

Klac AKLKEREYNKMLGKEVEESESDSEQQ---------SNPPVK-KKQRKSKYNLVSNNFKRL 382

Scer VKLKRRKVAEFLGSTSQISDTEFEHDPEASSGVVSSEQKPTAKRKGRKKYNLVSNNFRRL 437

Calb TKLEEGERKQLVAYMDSDEDDENRDGE--------VGIASVFE**SPTK**KTRMPVSNNFKRL 338

.**.. :: . . .. : .. : . **:**:**

Agos KLPTKNTRN--GRWRRR- 379

Klac NLPTKAGRNRASKWRGRR 400

Scer KLPKKNRFSN-GRWGRR- 453

Calb KINDPRSRRFKQRMRR-- 354

:: :

**C. Multiple sequence alignment between *S. cerevisiae* Swi5 protein and the closest homologue in related yeasts.**

Scer ------------------------------------------------------------

Agos -MAGEPWSIDPTALLHTHSNTGGDANARQGERVSLNLDYAAKDAVGELGLAVEAESEYFE 59

Klac MSSTEQWSNLIKNPYDVVDGNNGEISENMMDLLNSRNNSNNLGSVYGGQDYPLPDEAFEN 60

Calb -----------MHWKFSNFRKYHLSFHLNLFDLSLFFISFYCFPILYICFFNQVHSFRST 49

Scer ----------------------------------------------------MDTSNSWF 8

Agos LGTENYEDIDEFLTQELRDLDIPVAPAPGGCAGLVEPPACYPKRPEEEDVGIFASALDWT 119

Klac FNPVMDINIEEFLTKELRDLDIPVLPKDELSKLDLEMFPGNGLDWNVVNDNDASVGVGNV 120

Calb QPSLIMNKFDLFDDYSTKGSTIPLPNENFDQLFLSSEANDMEFLFNETLMGLQDLDVPSG 109

Scer DASKVQSLNFDLQTNSYYSNARGSDPSSYAIEGEYKTLATDDLGN-ILNLNYGETNEVIM 67

Agos ALEHVGSDSRTEPEQCVDRRTGRR**TP**SGTAIFGFSRHNKTLNIGAGGIAGDDPERGELLD 179

Klac NTEIPSI**TPRK**LMPGGGAAGNHKKKPSGTAIFGFAQHNKSLSIGSMTINLDE-KKQAFLQ 179

Calb YGIPQNTINNDFQH**TPNK**SKSHSRQYSGTAIFGFADHNKDLSINGVNNDLCKQSNKAINT 169

*. ** * .:. . .

Scer NEINDLNLPLGPLSDEKSVKVSTFSELIGNDWQSMNFDLENNSREVTLNATSLL-NENRL 126

Agos VKEKQSLPAMSSLIIKQQEELGLALERQKEMNRQLEEQLKITQLQQKQLQAVLN-QQESA 238

Klac QEQQKLLQAQQASQPAEIKMSETIMQQQKELQLALQRQKEMNEKLEDQLRMNKL-QQEQL 238

Calb QSV**SP**GELLKRSRGSQ**TPTP**TSALPDTAQDILDFNFEEKPILLLEEDELEEEKHKQQQRM 229

. . . : : : :::

Scer NQDSGMTVYQKTMSDKPHDEKKISMADNLLSTINKSEINKGFDRNLGELLLQQQQELREQ 186

Agos GRKLGGNTSIM**TP**LHHPQEYDSVLLASASRSGGYTFPILSEEEEDDDKEPPDTVLTGSRA 298

Klac QRALEAQQMTLNQVSSIPTT**TP**VRPETSIIITSNGKNGKYQFPPPSSDDSSQQRQMGERT 298

Calb MTQS**SPLK**RVT**TP**SQ**SP**FVQQPQTMKQRKPHKKTNEYIVANENPNSYKFPP**SPSP**TAKRQ 289

. . . .

Scer LRAQQEANKKLELELKQTQYKQ--QQLQATLENSDGPQFL**SPKR**KI**SP**ASENVEDVYANS 244

Agos TLNG**SPRK**QYLPLQETGALDTLGAV**SP**RGTAMDKLPPKTVPPISIERPLKLFPTGVQQQQ 358

Klac KLDE**SPNK**DVNKVYAVGAGNGLL**SP**FSRASVNG**SPHR**RRQHNYDGVENGNNRLAPPMNFS 358

Calb QYPPS**SP**IPYNPKSDSVGGNSYSAKYLQSLNKTQQIEYVDDIEPLLQEDNNNMKYIPIP- 348

. :. . .

Scer L**SP**MI**SP**PMSNTSFTG**SPSR**RNNRQKYCLQRKNSSGTVGPLCFQELNEGFNDSLI**SPKK**- 303

Agos HHQQQQQQQQQQQQQQQQQQQHGKQPCLIRQTFTVGSIDSGLGDQ**SP**L**SP**RHQLVAS**SP**K 418

Klac ISSTSTVGNNTEQLLKMSKYFQELTDNQSRGPSNCAVYNKKSSNRPPI**TP**QDQIYDNPKT 418

Calb ----VQEPMSYQKQKPV**TP**PLQSQNDSQQLEPLK**TP**QPQPKQQQQQQQPNNEQDKEFTAN 404

. . : . :. ...

Scer ----------IRSNPNENLSSKTKFI**TP**F**TP**KSRVSSATSNSANI**TP**NNLRLDFKINVED 353

Agos LAYESSV**TP**VLHRNSKSLSS**TP**STVFVPAEPESELSITSGKLQRPTTIGLGLHYHD-SQQ 477

Klac PSMNCNYKHTTRSDDGLETASSIDSNINNKNSHQNKHFARDSTISTASTIPMQSEDDHES 478

Calb ------------INFNTFLPPP**TP**PNLING**SP**DWNS**SP**EPH**SPSPGR**LQPPQQI**SP**IHQN 452

: .. . . . :.

Scer QESEYSEKPLGLGIELLGKPGP**SPTK**SVSLKSASVDIMPTIPGSVNN**TP**SVNKVSLSSSY 413

Agos PLVQTTNRPVSMGMLPSYKSKAFSGKESL**SP**PGYPQKYRFQQ**TP**LRD**SPQR**VMPIGSGRS 537

Klac EQTQQ-QPSLGLGITLNSRVQLKKPPQLQIMPMIPGSSET**TP**LKQKQ**SP**DRQFSNKLNSE 537

Calb LGAMGNNINFY**TP**MYYELPVQAEQPQPQPQPHQQQHQQQQHQPELQNTYQQIKHIQQQQQ 512

: . : .:: .

Scer IDQY**TP**RGKQLHFSSISENALGINAA**TP**HLKPPSQQARHREGVFNDLDPNVLTKNTDNEG 473

Agos SGSYPMNPNLFEMMK**SP**QLRPPGGAYYTHCRTRSDSTEYAEDRVPEFVHAK**SPSP**ILLSQ 597

Klac NMPVKHSFQH**TPTK**KLIFDKTVTTSLRPDDRKGQNARNIILASRDGEEDMKSGRLTNMNN 597

Calb MLQHQFHNQNNQLRQQHPNQFQNQNQNQNQNQTK**TP**YSQQSQF**SP**THSNFNL**SPAK**QLNS 572

: . . . . .

Scer DDNEENEPESRFVISE**TPSP**VLKSQSKYEGRS-------------------PQFGTHIKE 514

Agos EKFDKTHQFHGVSGTLGGSVMNGVGCKKVFDG-------------------HRPPGQYMG 638

Klac GNNDEPECEFVQAQ**TPSP**ILRSQERFDCIAE**SP**VHFPYNQHNHVDLGLGPNHNGIGLNTN 657

Calb NVGSMHL**SP**LKKQLPN**TP**TKQPPVTIEW**SP**VIS----------------**---**-PNSKQPL 612

. . .

Scer INTYTTN**SPSK**ITRKLTTLPRGSIDKYVKEMP-DKTFECLFPGCTKTFKRRYNIRSHIQT 573

Agos QSSIY**SPERSSPMK**KASNLPQGEIDQYIKKLQ-DKTFQCLYPECGKLFNRRYNIRSHIQT 697

Klac LNT**SP**MVSGGRNGKRYGMLPREQIDKYILEIG-PKQFQCKFKDCQKRFNRRYNARTHIQT 716

Calb HKQIKES**SPRR**RIKKTSLLPPGELDNYWTGPDEDKIYTCTYKNCGKKFTRRYNVRSHIQT 672

. . :: ** .:*:* * : * : * * *.**** *:****

Scer HLEDRPYSCDHPGCDKAFVRNHDLIRHKKSHQEKAYACP-CGKKFNREDALVVHRSRMIC 632

Agos HLEDRPFRCDHEGCTKAFVRNHDLIRHKKTHAEKTFTCP-CGKKFSREDALLTHRTRMIC 756

Klac HLCDRPYKCDFPGCQKAFVRNHDLLRHKKSHLEKGYSCAGCGKKFHSEDSLVKHQERKSH 776

Calb HLSDRPFGCQF--CPKRFVRQHDLNRHVKGHIEARYSKCPCGKEFARLDALRKHQDRNIC 730

** ***: *:. * * ***:*** ** * * * :: ***:* *:* *: *

Scer SGGKKYENVVIKR**SPRK**RGRPRKDGTSSVSS**SPIK**ENINKDHNGQLMFKLEDQLRRERSY 692

Agos VGGKKFDNIVIKK**SPRK**RGRPKKEGSSAHN-**SPVK**DVLARDYSGTVTLKMEKQLQKEPVN 815

Klac EGRLDEEDLCNQEQNHDLDYYDHLDS**SP**IRLNGGNQIRKPV**SPKK**VPNVIRENIQRGHTT 836

Calb VGGNKNVISKPTKKKGTNNTQQQLLKTDTVVERIEKQLLQEDKSVTEEFLMLQ------- 783

* . .. . : .: . :. : :

Scer DGNGTGIMV**SPMK**TNQR------------ 709

Agos PDLLQVPGS**TP**KAKITM**TP**VALRGFIT**SP** 844

Klac AALRVQEQLTYNQTTTGV----------- 854

Calb -----------------------------
